# Association between Serum Adipocyte Fatty Acid Binding Protein Level and Endothelial Dysfunction in Chronic Hemodialysis Patients

**DOI:** 10.3390/life12020316

**Published:** 2022-02-20

**Authors:** Hsin-Jou Fan, Chih-Hsien Wang, Bang-Gee Hsu, Jen-Pi Tsai

**Affiliations:** 1 Divisions of Nephrology, Hualien Tzu Chi Hospital, Buddhist Tzu Chi Medical Foundation, Hualien 97004, Taiwan; 0xliad53a4dfac58z%tzuchi@tzuchi.org (H.-J.F.); wangch33@tzuchi.com.tw (C.-H.W.); 2 School of Medicine, Tzu Chi University, Hualien 97004, Taiwan; 3 Division of Nephrology, Department of Internal Medicine, Dalin Tzu Chi Hospital, Buddhist Tzu Chi Medical Foundation, Chiayi 62247, Taiwan

**Keywords:** adipocyte fatty acid binding protein, endothelial dysfunction, hemodialysis, vascular reactivity index

## Abstract

Adipocyte fatty acid binding protein (A-FABP) is associated with atherosclerosis, and endothelial dysfunction is one of the reasons for adverse cardiovascular outcomes in patients undergoing hemodialysis (HD). This study investigated the correlation between serum A-FABP levels and endothelial function in HD patients. Fasting blood samples were collected from 90 HD patients. A-FABP levels were measured using a commercial enzyme immunoassay kit. Endothelial function was evaluated by a digital thermal monitoring test to measure vascular reactivity index (VRI). VRI < 1.0, 1.0 ≤ VRI < 2.0, and VRI ≥ 2.0 indicated poor, intermediate, and good vascular reactivity, respectively. In total, 14 (15.6%), 38 (42.2%), and 38 (42.2%) HD patients had poor, intermediate, and good VRI, respectively. Patients with poor VRI had lower pre-HD and post-HD body weight, body mass index, and serum creatinine level but higher serum A-FABP level (*p* = 0.001) than those with intermediate and good VRI. Log-transformed VRI (log-VRI) positively correlated with serum creatinine and negatively correlated with A-FABP by multivariate linear regression analysis. We concluded that A-FABP correlated with endothelial dysfunction in chronic HD patients.

## 1. Introduction

Cardiovascular disease (CVD) is the main cause of mortality in patients with chronic kidney disease (CKD), including those receiving dialysis [1]. Risk factors include traditional factors such as old age, hypertension (HTN), diabetes mellitus (DM), and CKD-specific factors such as inflammation, malnutrition, and endothelial damage or dysfunction [1,2]. In patients with predialysis, hemodialysis (HD), or peritoneal dialysis, a high prevalence of endothelial dysfunction was reported, which is associated with adverse long-term outcomes [2,3,4,5,6]. There is mounting evidence showing that inflammation, malnutrition, oxidative stress, and retention of uremic toxins are linked to the process of endothelial dysfunction [7,8,9,10,11]. 

Adipocyte fatty acid binding protein (A-FABP), a member of the FABP family, is primarily expressed in adipocytes and macrophages and has been linked to CVD as well as metabolic diseases such as obesity, DM, HTN, chronic inflammation, and atherosclerotic processes [12,13,14,15]. In addition to its association with tumor necrosis factor α, serum A-FABP level was found to be markedly higher in patients with critical sepsis and related to unfavorable survival outcomes [16]. There is evidence showing that circulating A-FABP could act as an adipokine and potentially as a biomarker linked to insulin resistance, DM, as well as CVD [17]. We recently reported that A-FABP was positively associated with endothelial dysfunction and arterial stiffness in patients with kidney transplantation and the geriatric population, respectively [18,19]. Because CVD is the leading cause of mortality in patients with CKD, and A-FABP is considered to play a vital role, but without reliable evidence in HD patients, we conducted this study to explore the relationship between A-FABP as well as other possible risk factors and endothelial function evaluated using a digital thermal monitoring test among HD patients.

## 2. Materials and Methods

### 2.1. Participants

From October 2017 to February 2018, with the approval of the Research Ethics Committee, Hualien Tzu Chi Hospital, Buddhist Tzu Chi Medical Foundation (IRB106-108-A), we enrolled 90 patients aged >20 years who were maintained on HD using standard bicarbonate dialysate (FX class dialyzer, Fresenius Medical Care, Bad Homburg, Germany) thrice a week for more than three months. Patients who refused to provide informed consent or had amputated limbs, active infection, malignancy, heart failure, and bedridden status were excluded. The basic characteristics, duration of receiving HD, and medical history that included DM, HTN, and medications used were reviewed from the medical charts.

### 2.2. Anthropometric Analysis and Biochemical Investigations

After measuring height and post-HD body weight, the body mass index (BMI) was calculated as post-HD body weight (kg) divided by height (m) squared [20]. About 5 mL blood sample was obtained before HD in each patient. After determining the hemoglobin level (Sysmex SP-1000i, Sysmex American, Mundelein, IL, USA), the remaining blood sample was centrifuged for biochemical analyses, including blood urea nitrogen, creatinine, albumin, total cholesterol, triglyceride, glucose, calcium, phosphorus, and alkaline phosphatase, using an autoanalyzer (SiemensAdvia 1800, Siemens Healthcare GmbH, Henkestr, Germany). The clearance of dialysis was measured as the fractional clearance index for urea (Kt/V) and urea reduction ratio using a formal, single-compartment dialysis urea kinetic model. Serum A-FABP (SPI-BIO, Montigny le Bretonneux, France) and intact parathyroid hormone (iPTH) (Abcam, Cambridge, MA, USA) levels were measured by a commercially available enzyme immunoassay or enzyme-linked immunosorbent assay kit [18,20,21].

### 2.3. Endothelial Function Measurements

The patients fasted overnight and abstained from using tobacco, alcohol, caffeine, and vasoactive medications, after which endothelial function was measured using a digital thermal monitoring device (VENDYS-II; Endothelix, Inc., Houston, TX, USA). After resting in a temperature-controlled room for 30 min, blood pressure cuffs were placed on the patient’s arm without arteriovenous vascular access, and skin temperature sensors were placed on bilateral index fingers. Digital thermal monitoring of both hands was performed with each step of 5-min stabilization, cuff inflation, and deflation as the blood pressure cuff was rapidly inflated to 50 mmHg greater than systolic blood pressure for 5 min and then rapidly deflated to invoke a reactive hyperemic response in the fingertip distally. The more the temperature rebounded, the better the vascular reactivity. Vascular reactivity index (VRI) was defined as the maximal temperature difference between the rebound curve and zero reactivity curves during the reactive hyperemia period, calculated using the VENDYS software. VRIs were found to be in the range of 0.0–3.5, where a VRI of <1.0 indicated poor vascular reactivity, a VRI of 1.0–1.9 indicated intermediate vascular reactivity, and a VRI of ≥2.0 indicated good vascular reactivity [18,22,23].

### 2.4. Statistical Analysis

Continuous variables were tested for normal distribution by the Kolmogorov–Smirnov test and expressed as mean ± standard deviation or median with interquartile range accordingly. Comparisons of continuous variables among groups (poor, intermediate, and good VRI) were performed by Kruskal–Wallis analysis (HD duration, VRI, triglyceride, glucose, alkaline phosphatase, and iPTH) or one-way analysis of variance according to results of the Kolmogorov–Smirnov test. Categorical variables were analyzed using the chi-square test and expressed as numbers and percentages. HD duration, VRI, triglyceride, glucose, alkaline phosphatase, and iPTH levels were log-transformed to fulfill normality for further analyses. Simple and multivariable linear stepwise regression analyses were conducted to examine the correlation between VRI and clinical and biochemical variables of HD patients. Univariate and multivariate logistic regression analysis was used to analyze the A-FABP level for vascular reactivity dysfunction (intermediate vascular reactivity and poor vascular reactivity) or poor vascular reactivity. Analyses were performed using SPSS for Windows (version 19.0; SPSS Inc., Chicago, IL, USA). G*power was used to calculate the sample size and power (Franz Faul, Iniversitat Kiel, Germany; 3.1.9.6). A *p*-value of <0.05 was considered statistically significant.

## 3. Results

Table 1 shows the clinical characteristics and medications used of 90 HD patients. Of the 90 HD patients, 14 (15.6%) had poor VRI, 38 (42.2%) had intermediate VRI, and 38 (42.2%) had good VRI. There were significant between-group differences in lower pre-HD body weight (*p* = 0.016), post-HD body weight (*p* = 0.013), BMI (*p* = 0.034), and serum creatinine level (*p* = 0.010), and higher serum A-FABP levels (*p* = 0.001) were found in groups with worse VRI. Regarding comorbid conditions, 52 patients (57.8%) were identified to have HTN, and 49 patients (54.4%) had DM. No significant differences were observed in the following factors among the different groups: gender, blood pressure, presence of HTN or DM, and use of antihypertensive drugs and anti-lipid drugs.

**Figure 1:**
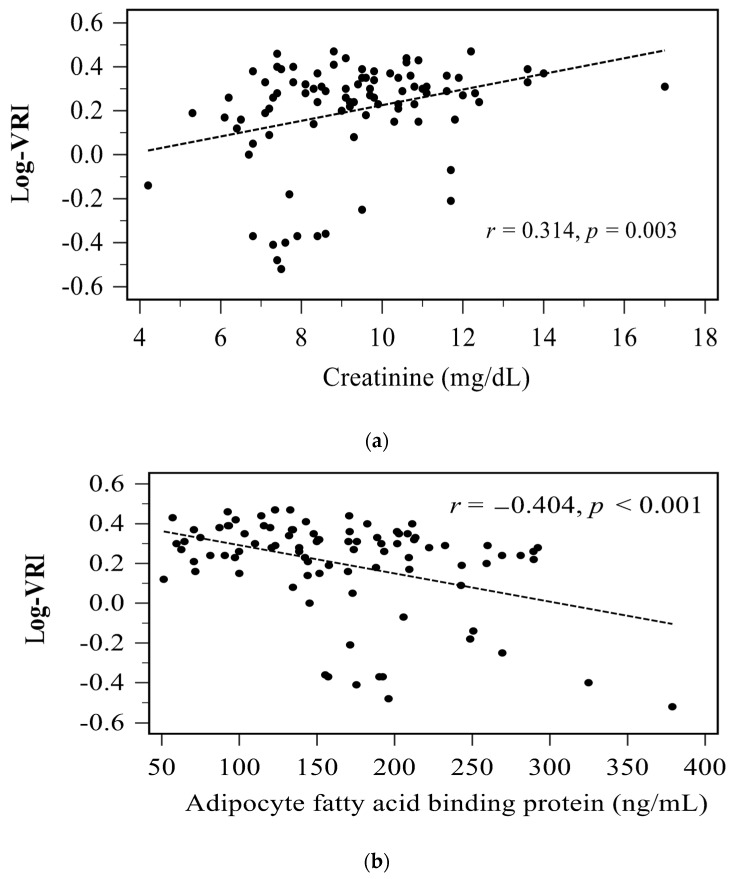
Relationships between log-transformed vascular reactive index (log-VRI) and (**a**) creatinine, (**b**) adipocyte fatty acid binding protein level among 90 hemodialysis patients.

The correlations between the clinical characteristics and serum log-transformed VRI (log-VRI) values of the 90 HD patients are presented in Table 2. Serum A-FABP level (*r* = −0.404, *p* < 0.001) correlated negatively with log-VRI values, whereas pre-HD body weight (*r* = 0.278, *p* = 0.008), post-HD body weight (*r* = 0.284, *p* = 0.007), BMI (*r* = 0.242, *p* = 0.022), and serum creatinine level (*r* = 0.314, *p* = 0.003) correlated positively with log-VRI values in HD patients. Multivariate forward stepwise linear regression analysis of the variables that were significantly associated with log-VRI revealed that serum A-FABP level (β = −0.349, adjusted R^2^ change = 0.153, *p* = 0.001) and serum creatinine level (β = 0.232, adjusted R^2^ change = 0.042, *p* = 0.020) were significantly and independently associated with log-VRI values in HD patients. Two-dimensional scatter plots of VRI values with serum creatinine level and serum A-FABP level among patients undergoing HD are presented as Figure 1a,b, respectively.

Compared to patients with good vascular reactivity index, patients with vascular reactivity dysfunction (intermediate and poor vascular reactivity groups) (odds ratio (OR) = 1.010; 95% confidence interval (CI) = 1.002–1.018; *p* = 0.011) and poor vascular reactivity index (OR =1.014, 95% CI = 1.003–1.025, *p* = 0.011) showed A-FABP was positively and independently associated with poor vascular reactivity, respectively, by univariate and multivariate logistic regression analysis adjusted data for factors including pre-HD body weight, post-HD body weight, BMI, creatinine, and A-FABP levels (Table 3).

## 4. Discussion

This study demonstrated that after adjusting for confounders, serum A-FABP level correlated negatively with VRI, and creatinine level correlated positively with VRI measured by digital thermal monitoring in HD patients.

Along with traditional risk factors for CVD in patients with advanced CKD, including those on dialysis, endothelial dysfunction is additionally considered as a progressive factor [1,3,6,8]. Evidence showed that nontraditional risk factors such as hyperphosphatemia could modulate the process of endothelial dysfunction as demonstrated by increased circulating levels of intracellular adhesion molecule-1 and vascular adhesion molecule-1, which are markers of cardiovascular (CV) events in patients with CKD [24]. Flow-mediated dilation and nitroglycerin-mediated dilation were found to be impaired by mechanisms including endothelium-dependent vasodilation and decreased bioavailability of nitric oxide [4], and the endogenous inhibitor of nitric oxide synthase could be a potential biomarker of endothelial dysfunction along with C-reactive protein to predict all-cause and CV mortality in HD patients [25]. Accumulating evidence shows that CKD-related endothelial dysfunction is associated with the development of atherosclerosis, which could be attributed to uremic toxins through oxidative stress or inflammation [11,26]. For instance, indoxyl sulfate, a type of uremic toxin, was recently found to be associated with VRI in patients with stage 3 to 5 CKD [27]. In this study, as potential nontraditional risk factors, we additionally found that creatinine, BMI, and body weight, either predialysis or postdialysis, correlated positively with VRI; in particular, creatinine was independently associated with VRI after adjusting for confounders. Similar to previous studies conducted on patients undergoing peritoneal dialysis, who had significantly lower flow-mediated dilation and higher soluble intercellular adhesion molecule-1 levels in the malnourished group, a positive correlation was detected among lean body mass, body weight, albumin level, and flow-mediated dilation [7]. However, another study reported no difference in asymmetric dimethylarginine, which is a marker of endothelial dysfunction, between lean and overweight patients undergoing peritoneal dialysis [28]. Considering all these studies together, although there was no evidence indicating a conclusive relationship between nutritional status and endothelial function, we herein found that creatinine, which was indicative of muscle mass, correlated positively with endothelial function.

After its discovery in 1972, A-FABP, a small cytosolic polypeptide abundantly expressed in tissues with active lipid metabolism, such as the heart and liver or cells specialized for lipid storage, trafficking, and signaling such as adipocytes and macrophages, was well known to be associated with several metabolic disorders and CVD [12,13]. In the study of Tanno-Sobetsu, A-FABP was found to be negatively correlated with peak myocardial velocity during early diastole, which was indicative of diastolic dysfunction [15]. In a longitudinal study, A-FABP level was higher in morbid obese than in lean women and correlated positively with BMI, homeostasis model assessment of insulin resistance, tumor necrosis factor receptors, and C-reactive protein [13]. Follow-up of women with a known history of gestational diabetes showed that serum A-FABP levels were independently associated with impaired glucose tolerance, and together with BMI and abdominal fat distribution, it might increase the risk of progression to DM [29]. Furthermore, A-FABP was positively related to renal dysfunction presented as elevated serum creatinine, fasting glucose, tumor necrosis factor α, and negatively associated with albumin and considered to be a potential prognostic biomarker associated with unfavorable CV outcomes in patients with critical illness [16]. In patients undergoing HD and those with coronary artery disease, A-FABP level was markedly higher than that of normal control as well as independently correlated with higher adverse CV events during long-term follow-up [30,31]. In in vivo and in vitro studies, blockade of the expression of A-FABP by an inhibitor or by the knockout method revealed that A-FABP might contribute to endothelial dysfunction or atherosclerosis through alteration of inflammatory cytokine production or modulation of the activation of endothelial nitric oxide synthase [32,33]. An in vitro study using human coronary endothelial cells from angiographically proven coronary artery disease revealed that A-FABP could enhance the expression of intracellular adhesion molecule-1, vascular adhesion molecule-1, and P-selectin through the ERK/JNK/STAT-1 signaling pathway as well as by suppressing the activation of endothelial nitric oxide [34]. Moreover, A-FABP was found to have an inhibitory role in the expression of insulin-mediated endothelial nitric oxide synthase and NO production by inhibiting the activation of insulin receptor substrate 1 and Akt [35]. In addition, mounting evidence has shown that serum A-FABP levels were associated with endothelial dysfunction, peripheral arterial disease, or arterial stiffness [18,19,20,21,36]. In patients with DM and kidney transplantation, circulating A-FABP levels were independently associated with endothelial dysfunction measured by reactive hyperemia index or VRI, respectively [18,36]. Considering all these studies together, we detected a significant negative correlation between serum A-FABP levels and VRI of HD patients, but the detailed mechanisms require further studies.

There were limitations of this study. Firstly, this was a cross-sectional design study. Secondly, there was a limited number of HD patients, which may have affected the statistical significance. Only 90 HD participants were enrolled in this study, and the power to predict endothelial function was only 0.73. Third, this study did not measure the B-type natriuretic peptide or hydration status of patients. However, the findings of our previous studies [37,38] suggest that this impact may be limited, as the influence of overhydration in A-FABP or VRI measurements was found to be trivial. Hence, the causal relationship between serum A-FABP levels and endothelial function of HD patients should be investigated with more patients in longitudinal studies.

## 5. Conclusions

This study elucidated the risk factors for endothelial dysfunction measured by VRI and showed that along with serum lower creatinine levels, higher A-FABP levels could be a potential biomarker of endothelial dysfunction in HD patients.

## Figures and Tables

**Table 1:** Clinical characteristics of all HD patients and among groups.

Characteristics	All Patients (*n* = 90)	Good Vascular Reactivity (*n*= 38)	Intermediate Vascular Reactivity (*n* = 38)	Poor Vascular Reactivity (*n* = 14)	*p*-Value
Age (years)	59.97 ± 13.48	58.01 ± 12.88	63.06 ± 14.44	56.92 ± 11.36	0.173
Height (cm)	162.78 ± 8.03	162.84 ± 7.73	164.04 ± 8.80	159.21 ± 5.74	0.158
Pre-HD body weight (kg)	65.96 ± 14.79	67.23 ± 15.67	68.45 ± 14.27	55.74 ± 9.13	0.016 *
Post-HD body weight (kg)	63.37 ± 14.29	64.72 ± 15.15	65.76 ± 13.68	53.25 ± 8.95	0.013 *
Body mass index (kg/m^2^)	24.73 ± 4.37	25.18 ± 4.66	25.29 ± 4.14	21.95 ± 3.24	0.034 *
HD duration (months)	44.34 (26.55–81.90)	40.92 (25.59–92.79)	40.74 (20.01–80.04)	51.90 (40.02–87.57)	0.358
Vascular reactivity index	1.89 (1.45–2.23)	2.29 (2.11–2.50)	1.70 (1.48–1.85)	0.44 (0.40–0.66)	<0.001 *
SBP (mmHg)	150.82 ± 26.95	153.13 ± 26.97	148.39 ± 25.84	151.14 ± 31.11	0.960
DBP (mmHg)	82.24 ± 15.15	83.87 ± 15.07	79.74 ± 14.70	84.64 ± 16.69	0.406
Hemoglobin (g/dL)	10.24 ± 1.30	10.26 ± 1.29	10.24 ± 1.33	10.19 ± 1.37	0.989
Albumin (g/dL)	4.18 ± 0.44	4.25 ± 0.48	4.09 ± 0.40	4.24 ± 0.40	0.241
Total cholesterol (mg/dL)	158.99 ± 38.59	160.66± 37.58	155.16 ± 39.51	164.86 ± 40.57	0.686
Triglyceride (mg/dL)	117.00 (77.75–202.75)	111.00 (77.25–191.50)	127.50 (80.00–207.50)	95.50 (62.25–188.75)	0.820
Glucose (mg/dL)	120.00 (94.75–181.75)	119.00 (100.00–175.00)	133.00 (97.75–195.75)	103.50 (77.50–136.50)	0.109
Alkaline phosphatase (U/L)	75.00 (61.50–104.25)	68.50 (57.75–96.00)	75.50 (62.25–110.75)	95.00 (69.50–125.50)	0.167
Blood urea nitrogen (mg/dL)	60.09 ± 15.19	60.82 ± 13.93	58.95 ± 15.60	61.21 ± 18.12	0.831
Creatinine (mg/dL)	9.28 ± 2.11	9.95 ± 2.16	9.05 ± 1.90	8.07 ± 1.94	0.010 *
Total calcium (mg/dL)	9.11 ± 0.75	9.13 ± 0.73	9.02 ± 0.80	9.27 ± 0.68	0.550
Phosphorus (mg/dL)	4.75 ± 1.44	4.95 ± 1.46	4.57 ± 1.33	4.72 ± 1.71	0.521
iPTH (pg/mL)	216.00 (98.88–1482.15)	291.35 (161.25–471.73)	161.50 (67.93–482.65)	207.65 (93.06–662.45)	0.232
A-FABP (ng/mL)	164.79 ± 67.22	140.61 ± 48.26	169.14 ± 71.93	218.64 ± 48.48	0.001 *
Urea reduction rate	0.73 ± 0.05	0.73 ± 0.05	0.72 ± 0.05	0.75 ± 0.06	0.288
Kt/V (Gotch)	1.32 ± 0.21	1.31 ± 0.19	1.29 ± 0.20	1.40 ± 0.26	0.262
Female, *n* (%)	34 (37.8)	14 (36.8)	12 (31.6)	8 (57.1)	0.238
Diabetes mellitus, *n* (%)	49 (54.4)	19 (50.0)	24 (63.2)	6 (42.9)	0.329
Hypertension, *n* (%)	52 (57.8)	18 (47.4)	24 (63.2)	10 (71.4)	0.201
CAD, *n* (%)	15 (16.7)	4 (10.5)	9 (23.7)	2 (14.3)	0.296
PAD, *n* (%)	4 (4.4)	1 (2.6)	2 (5.3)	1 (7.1)	0.743
ARB use, *n* (%)	42 (46.7)	15 (39.5)	18 (47.4)	9 (64.3)	0.280
β-blocker use, *n* (%)	19 (21.1)	6 (15.8)	10 (26.3)	3 (21.4)	0.531
CCB use, *n* (%)	37 (41.1)	16 (42.1)	13 (34.2)	8 (57.1)	0.325
α-adrenergic blockers, *n* (%)	30 (33.3)	11 (28.9)	13 (34.2)	6 (42.9)	0.633
Statin use, *n* (%)	21 (23.3)	9 (23.7)	10 (26.3)	2 (14.3)	0.660
Fibrate use, *n* (%)	18 (20.0)	10 (26.3)	5 (13.2)	3 (21.4)	0.354

HD—hemodialysis; SBP—systolic blood pressure; DBP—diastolic blood pressure; iPTH—intact parathyroid hormone; A-FABP—adipocyte fatty acid binding protein; Kt/V—fractional clearance index for urea; CAD—coronary artery disease; PAD—peripheral artery disease; ARB—angiotensin receptor blocker; CCB—calcium channel blocker. * *p* < 0.05 was considered statistically significant.

**Table 2:** Correlation of vascular reactivity index levels and clinical variables by simple or multivariable linear analyses among 90 hemodialysis patients.

Variables	Log Transformed Vascular Reactivity Index						
	Simple Regression			Multivariable Regression			
	*r*	95% CI	*p*-Value	Beta Coefficient	95% CI	Adjusted R^2^ Change	*p*-Value
Age (years)	−0.004	−0.004–0.004	0.971	—	—	—	—
Height (cm)	0.196	−0.001–0.012	0.063	—	—	—	—
Pre−HD body weight (kg)	0.278	0.001–0.008	0.008 *	—	—	—	—
Post−HD body weight (kg)	0.284	0.001–0.008	0.007 *	—	—	—	—
Body mass index (kg/m^2^)	0.242	0.002–0.024	0.022 *	—	—	—	—
Log−HD duration (months)	−0.134	−0.217–0.048	0.208	—	—	—	—
Systolic blood pressure (mmHg)	0.088	−0.001–0.003	0.407	—	—	—	—
Diastolic blood pressure (mmHg)	0.049	−0.003–0.004	0.646	—	—	—	—
Hemoglobin (g/dL)	0.040	−0.031–0.046	0.709	—	—	—	—
Albumin (g/dL)	0.054	−0.058–0.144	0.613	—	—	—	—
Total cholesterol (mg/dL)	−0.049	−0.002–0.001	0.646	—	—	—	—
Log−Triglyceride (mg/dL)	0.071	−0.115–0.231	0.504	—	—	—	—
Log−Glucose (mg/dL)	0.080	−0.158–0.348	0.456	—	—	—	—
Log−ALP (U/L)	−0.161	−0.437–0.057	0.129	—	—	—	—
Blood urea nitrogen (mg/dL)	0.073	−0.002–0.004	0.491	—	—	—	—
Creatinine (mg/dL)	0.314	0.013–0.058	0.003 *	0.232	0.004–0.048	0.042	0.020 *
Total calcium (mg/dL)	−0.023	−0.075–0.060	0.827	—	—	—	—
Phosphorus (mg/dL)	0.137	−0.012–0.057	0.198	—	—	—	—
Log−iPTH (pg/mL)	0.071	−0.059–0.119	0.504	—	—	—	—
A−FABP (ng/mL)	−0.404	−0.002–−0.001	<0.001 *	−0.349	−0.002–0.001	0.153	0.001 *
Urea reduction rate	−0.159	−1.652–0.224	0.134	—	—	—	—
Kt/V (Gotch)	−0.179	−0.443–0.033	0.091	—	—	—	—

Data of HD duration, VRI, triglyceride, glucose, ALP, and iPTH showed skewed distribution and therefore were log-transformed before analysis. Analysis of data was performed using the simple linear regression analyses or multivariable stepwise linear regression analysis (adapted factors were pre-HD, post-HD body weight, body mass index, creatinine, and A-FABP). CI—confidence interval; HD—hemodialysis; ALP—alkaline phosphatase; iPTH—intact parathyroid hormone; A-FABP—adipocyte fatty acid binding protein; Kt/V—fractional clearance index for urea. * *p* < 0.05 was considered statistically significant.

**Table 3:** Univariate and multivariate logistic regression analysis for vascular reactivity dysfunction (intermediate vascular reactivity and poor vascular reactivity) or poor vascular reactivity among 90 hemodialysis patients.

Model	A-FABP (Per 1 ng/mL of Increase) for Vascular Reactivity Dysfunction		A-FABP (Per 1 ng/mL of Increase) for Poor Vascular Reactivity	
	OR (95% CI)	*p*-Value	OR (95% CI)	*p*-Value
Crude model	1.011 (1.003–1.018)	0.005 *	1.014 (1.005–1.024)	0.003 *
Adjusted model	1.010 (1.002–1.018)	0.011 *	1.014 (1.003–1.025)	0.011 *

Adjusted model: pre-HD body weight, post-HD body weight, body mass index, creatinine, and A-FABP. HD—hemodialysis; A-FABP—adipocyte fatty acid binding protein; OR—odds ratio; CI—confidence interval. * *p* < 0.05 was considered statistically significant.
